# BAG3‐positive pancreatic stellate cells promote migration and invasion of pancreatic ductal adenocarcinoma

**DOI:** 10.1111/jcmm.14352

**Published:** 2019-05-22

**Authors:** Ye Yuan, Jing‐Yi Jiang, Jia‐Mei Wang, Jia Sun, Chao Li, Bao‐Qin Liu, Jing Yan, Xiao‐Na Meng, Hua‐Qin Wang

**Affiliations:** ^1^ Department of Biochemistry & Molecular Biology China Medical University Shenyang China; ^2^ Key Laboratory of Cell Biology, Ministry of Public Health, and Key Laboratory of Medical Cell Biology, Ministry of Education China Medical University Shenyang China; ^3^ Cancer Hospital of China Medical University Liaoning Province Shenyang P R China; ^4^ Liaoning Cancer Hospital & Institute Liaoning Province Shenyang P R China

**Keywords:** BAG3, invasion, microenvironments, PDACs, PSCs

## Abstract

BAG3 is constitutively expressed in multiple types of cancer cells and its high expression is associated with tumour progression and poor prognosis of PDAC**.** However, little is known about the role of BAG3 in the regulation of stromal microenvironment of PDAC. The current study demonstrated that beside PDAC tumour cells, BAG3 was also expressed in some activated stroma cells in PDAC tissue, as well as in activated PSCs. In addition, the current study demonstrated that BAG3 expression in PSCs was involved in maintenance of PSCs activation and promotion of PDACs invasion via releasing multiple cytokines. The current study demonstrated that BAG3‐positive PSCs promoted invasion of PDACs via IL‐8, MCP1, TGF‐β2 and IGFBP2 in a paracrine manner. Furthermore, BAG3 sustained PSCs activation through IL‐6, TGF‐β2 and IGFBP2 in an autocrine manner. Thereby, the current study provides a new insight into the involvement of BAG3 in remodelling of stromal microenvironment favourable for malignant progression of PDAC, indicating that BAG3 might serve as a potential target for anti‐fibrosis of PDAC.

## 1 INTRODUCTION

Pancreatic ductal adenocarcinoma (PDAC), one of the most difficult fortresses to cross in medicine, remains the fourth leading cause of cancer‐related death worldwide.^1^, ^2^ Despite encouraging progress in our understanding of molecular pathogenesis of pancreatic cancer and advances in the development of new chemotherapeutic agents, the prognosis of PDAC is dismal with a 5‐year survival rate of less than 5%.^3^ This poor prognosis is due to difficulty in early detection, high prevalence of metastasis and resistance to current chemotherapies. Therefore, it is of great importance to clarify the mechanism underlying pancreatic cancer progression and to identify novel targets for treatment.

A dense desmoplastic stromal response surrounding the islands of cancer cells is the typical histological features of PDAC. Increasing evidence shows that pancreatic desmoplastic stroma plays a pivotal role in tumourigenesis, metastasis and resistance to chemotherapy of PDAC.^4^, ^5^, ^6^ The stromal tissue sometimes comprises up to 80% of tumour mass and is characterized by extensive fibrosis, hypovascularity and hypoxia.^7^, ^8^ The stroma of PDAC is composed of cellular components such as pancreatic stellate cells (PSCs), carcinoma‐associated fibroblasts (CAFs) and immune cells and acellular components extracellular matrix (ECM).^9^, ^10^ These complex and heterogeneous stromal components constitute a sophisticated microenvironment that facilitates tumour growth and metastasis. Complex interactions between stromal cells and pancreatic cancer cells exert influences upon each other. On one hand, tumour cells secrete pro‐inflammatory soluble factors such as TGF‐β1, PDGF, TNF‐α and IL‐1/6, which recruit and activate PSCs/CAFs. On the other hand, activated PSC/CAFs secrete large amounts of extracellular matrix (ECM) proteins and signalling factors to remodel tumour microenvironment‐assisting malignant progression of PDAC.^11^ Based on the key role of tumour stroma, a number of stromal‐targeting strategies in PDAC have been developed. However, so far none of the stromal‐ablation therapeutic strategies have improved patient survival and some of them even had the adverse effect,^12^, ^13^, ^14^ suggesting that more studies are needed to further decipher the complexity of PDAC tumour‐stromal interactions.

Bcl2‐associated athanogene (BAG) 3 belongs to BAG family of co‐chaperones that interact with the ATPase domain of the heat shock protein 70 (Hsp70) via the carboxyl terminal BAG domain.^15^ Besides, BAG3 has multiple domains such as WW domain, proline‐rich (PxxP) domain and IPV (Ile‐Pro‐Val) motifs, providing the structural basis for interactions with other partners. By interacting with different partners, BAG3 protein participates in modulating a variety of biological processes including anti‐apoptosis, autophagy, cytoskeleton organization and cell motility. BAG3 is constitutively expressed in many cancer tissues, including pancreatic ductal adenocarcinoma cells (PDACs),^16^ melanomas,^17^ colorectal carcinomas^18^ and thyroid carcinomas,^19^ contributing to tumour growth, invasiveness and resistance to therapy. More recent literature shows that BAG3 can be secreted by pancreatic cancer cells.^20^, ^21^ The secreted BAG3 can bind and activate stromal macrophages to promote pancreatic cancer cells growth in turn. However, involvement of BAG3 in remodelling of stromal microenvironment in PDAC is not fully studied.

In the current study, we observe that conditioned media from BAG3‐overexpression PSCs facilitate migration and invasion of PDACs and promote proliferation and migration of PSCs. Furthermore, we demonstrate that ectopic expression of BAG3 in PSCs remodels stromal microenvironment of PDACs through mediating secretion of some cytokines/chemokines. These cytokines/chemokines exert an influence on PDACs and PSCs in a paracrine and autocrine manner respectively. Thereby, we provide a new insight into the involvement of BAG3 in interaction between PDACs and PSCs, indicating that BAG3 might serve as a potential target for anti‐fibrosis of PDAC.

## 2 MATERIALS AND METHODS

### 2.1 Patients and tissue samples

In this study, we enroled 30 patients with PDAC who had undergone pancreatic surgery at Liaoning Cancer Hospital & Institute between July 2016 and July 2018. Eligible patients were the participants diagnosed pathologically with PDAC by two qualified pathologists according to the WHO classification. Those who accepted radiotherapy, chemotherapy or other treatments before surgery were excluded from this study. All tissue specimens were processed in formalin fixation for 24 hours and then embedded in paraffin. The protocol was authorized by the Ethics Committee of China Medical University and the informed consent was obtained from each participant.

### 2.2 Cell culture

The human pancreatic cancer cell lines BxPC3 and SW1990 were obtained from ATCC and cultured in Dulbecco's Modified Eagle's Medium (DMEM) supplemented with 10% foetal bovine serum (FBS). The human primary pancreatic stellate cell line HPanSteC was purchased from ScienCell Research Laboratories (California, USA) cultured with Stellate Cell Medium (ScienCell, Cat #5301, 500 mL of basal medium supplemented with 10 mL of FBS, 5 mL of Stellate Cell Growth Supplement and 5 mL of penicillin/streptomycin solution) at a cell density of 5 × 10^3^/cm^2^ as the supplier recommended. Cell culture medium was changed every three days until the culture reached approximately 90%, then the culture was passaged. The human primary pancreatic stellate cell line HPanSteC was used within five to six passages from initiation. All cell lines were grown at 37°C in a 5% CO_2_ atmosphere.

### 2.3 Recombinant lentivirus infection

To explore the impact of BAG3 on stromal microenvironment of PDACs, retroviral vectors carrying BAG3 gene were constructed (GeneChem Co., Ltd., Shanghai, China). Human pancreatic stellate cell line HPanSteC was infected with recombinant virus‐expressing BAG3 or control virus.

### 2.4 Immunohistochemistry

Briefly, 4‐micrometre sections were cut from Paraffin‐embedded tissue blocks, mounted on poly‐L‐lysine‐coated slides, deparaffinized and hydrated. After being boiled in citric acid buffer for 90 seconds and blocked by hydrogen peroxide and normal goat serum, sections were incubated with anti‐BAG3 antibody (GeneTex) and anti‐α‐SMA antibody (Abcam) for 2 hours at 25°C. Subsequent to incubation with secondary antibodies for one hour, sections were detected using the Streptavidin‐Peroxidase complex (component C and D, UltraSensitiveTM SP [Goat] IHC Kit 9719) and diaminobenzidine (DAB Kit‐1031, Maixin Inc, Fujian, China). Finally, the sections were counterstained with haematoxylin and then dehydrated and mounted.

### 2.5 Collection of conditioned media

HPanSteC cells (5 × 10^6^ cells) were cultured in T‐175 flasks in Stellate Cell Medium. On the next day, the cells were washed two to three times with PBS until no suspended dead cells were left. Then the cells were cultured in Stellate Cell Medium containing 1% FCS for additional 72 hours. Thereafter, the supernatants were collected, aliquoted into 1.5‐mL tubes and stored at −80°C until usage.

### 2.6 Growth and migration assays using real‐time cellular analysis (RTCA)

Real‐time monitoring of cell proliferation and migration were performed with the xCELLigence system (ACEA Biosciences, San Diego, CA) according to the manufacturer's directions. To evaluate cell growth, 2  ×  10^4^ cells per well were seeded in quadruplicate on E‐plate with bottom surfaces covered with microelectrode sensors (ACEA Bioscience) and the electrical impedance in each well was measured continuously. Real‐time changes in electrical impedance were expressed as ‘cell index.’ For cell migration experiments, CIM‐plate (ACEA Biosciences, San Diego, CA) was adopted. CIM‐plate is composed of upper and lower chambers separated by an 8‐μm microporous membrane. About 5  ×  10^4^ cells per well were added in quadruplicate to the upper chambers. Migration was measured as the relative impedance change (cell index) across microelectronic sensors integrated into the bottom side of the membrane.

### 2.7 Migration and invasion assays by transwell

Cell migration/invasion assays were measured in Corning 3422 transwell permeable support chambers with 8‐mm pore filter inserts in 24‐well plates (Corning Incorporated Life Sciences, Munich, Germany). The invasion assay shared the same procedures, except that the filter inserts were pre‐coated with Matrigel at a 1:4 dilution in DMEM. Briefly, 600 µL DMEM containing 10% FBS was added to the lower chamber. A total of 100 µL cells in serum‐free DMEM were seeded into the top chamber (3 × 104 cells/well). After incubation for 24 hours, the cells on the upper surface were removed using a cotton swab. The migrated/invaded cells on the lower surfaces of inserts were fixed in methanol and stained with crystal violet. The migrated/invaded cells were counted in 10 representative microscopic fields and photographed.

### 2.8 Human cytokine antibody array

BAG3 overexpressed human pancreatic stellate cell HPanSteC and control cells were cultured in DMEM (high glucose) with 0.2％FBS for 72 hours. Then the cell culture supernatant was obtained by centrifugation. The conditioned media were analysed with RayBio® Human Cytokine Antibody Array C‐Series C5 (RayBiotech, USA). Briefly, the membranes were incubated in conditioned medium overnight at 4°C after half an hour of incubation with blocking buffer. Membranes were washed three times with wash buffer Ⅰ and Ⅱ respectively, followed by incubation with biotinylated antibodies mixture overnight at 4°C. Then, membranes were incubated with horseradish peroxidase‐conjugated streptavidin overnight at 4°C after washing. Finally, after full washing, the membranes were detected using detection buffer and scanned with an imaging system (Tanon‐4200; Tanon Science & Technology Co., Ltd).

### 2.9 Enzyme‐linked immunosorbent assays

In order to quantify the content of some cytokines/chemokines in supernatant of HPanSteC cells, enzyme‐linked immunosorbent assays (ELISA) were conducted according to the provided instructions. ELISA kits for all factors were purchased from RayBiotech.

### 2.10 RNA isolation and Quantitative reverse transcriptase PCR

RNA from cultured cells was isolated using RNeasy® Mini Kit (Qiagen), followed by cDNA synthesis using GoScript^TM^ Reverse Transcription System (Promega). Quantitative reverse transcriptase PCR was performed with GoTaq® qPCR Master Mix (Promega) on the ABI prism 7000 sequence detection system (Applied Biosystems, Eugene, OR). The results for each sample were normalized to the 18SrRNA. All assays were conducted at least three times.

### 2.11 Western blot analysis

Cells were lysed by RIPA lysis buffer (Thermo Fisher) supplemented with a protease inhibitor cocktail (Sigma‐Aldrich). Protein amount was determined using the BCA protein assay kit (Thermo Fisher). Twenty micrograms of total protein was separated on 10% SDS‐PAGE and transferred to PVDF membrane (Millipore). The membranes were blocked in 5% skimmed milk in Tris‐buffered saline buffer with 1% Tween‐20 (TBST) for 1 hour at room temperature, followed by incubation with primary antibodies overnight at 4°C. Subsequently, the membranes were subjected to HRP‐conjugated secondary antibodies, detected by enhanced chemiluminescence (ECL) solution (Millipore). Immunoreactivity on the membrane was visualized with an imaging system (Tanon‐5800, Tanon Science & Technology Co., Ltd). The antibodies in this study included: BAG3 (GeneTex), SMA (Abcam), GROα (GeneTex), GROβ (GeneTex), GROγ (Novus Biologicals), MCP1 (CST), CXCL6 (Invitrogen), IGFBP2 (CST), TIMP1 (Abcam), TIMP2 (Abcam), GAPDH (Sigma‐Aldrich).

### 2.12 Statistical analysis

SPSS (16.0) software (SPSS, Chicago, IL) was adopted for statistical analysis. All results were presented as the mean ± standard deviation. Data were analysed by Student's t‐test. All tests were two‐tailed and *P* < 0.05 was considered statistically significant.

## 3 RESULTS

### 3.1 BAG3 expression is up‐regulated in activated PSCs

**Figure 1:**
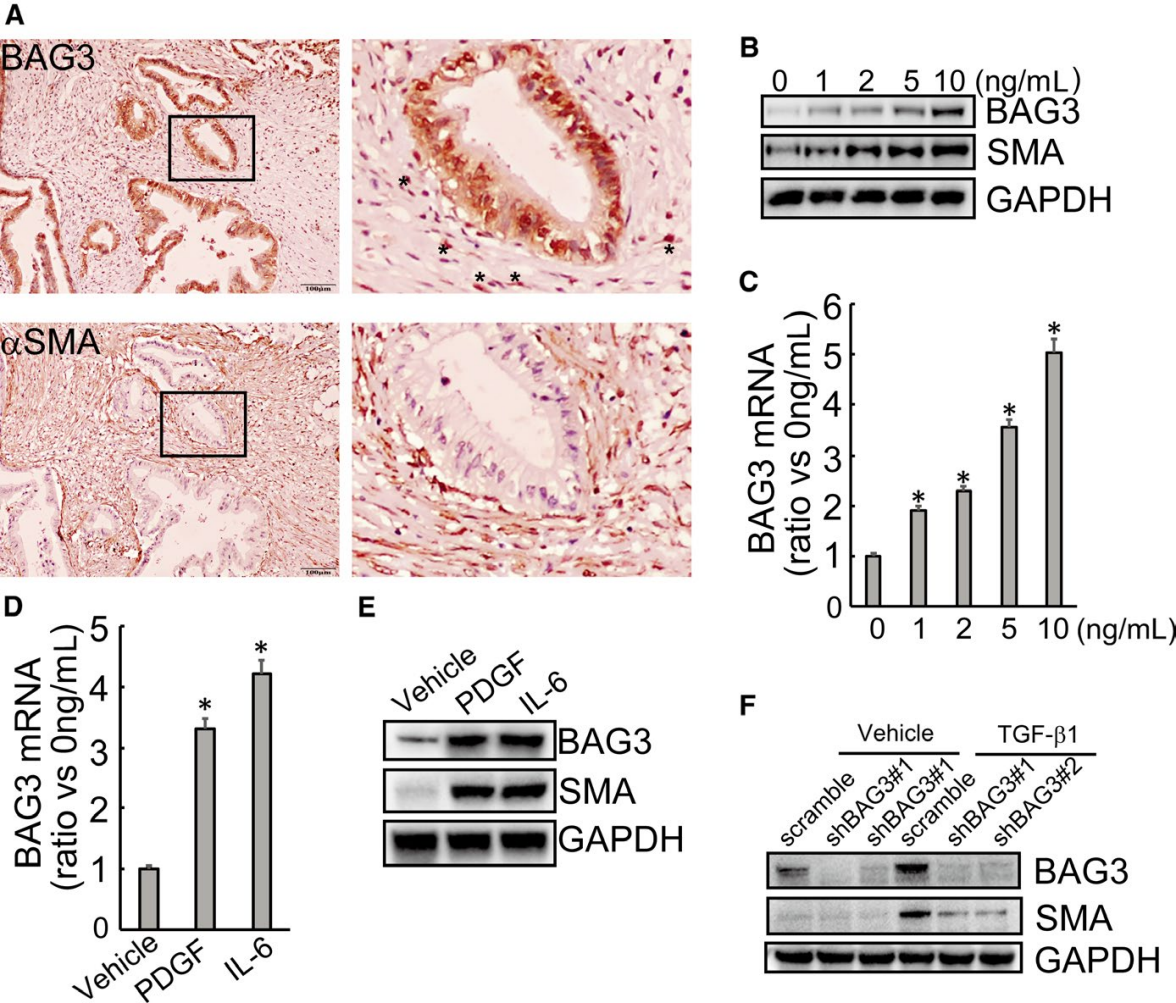
BAG3 is highly expressed in activated pancreatic stellate cells. (A) Representative immunohistochemical staining of BAG3 (upper left) and α‐SMA (upper right) in human PDAC tumour cells and stroma. The lower images show the selected boxed area of upper left. (B) HPanSteC cells were treated with TGF‐β1 (1, 2, 5 and 10 ng/mL) for 48 hours and the protein levels of BAG3 and α‐SMA were analysed by Western blot. (C) HPanSteC cells were treated with the indicated concentrations of TGF‐β1 for 24 h, the BAG3 mRNA level was detected by RT‐qPCR. (D and E) HPanSteC cells were stimulated with PDGF and IL6, mRNA level and protein level of BAG3 were analysed by RT‐qPCR and Western blotting respectively. (F) HPanSteC cells were infected with lentivirus containing shRNAs against BAG3 (shBAG3) for 48 h, then treated with 10 ng/mL TGF‐β1 for additional 24 h. Western blotting was performed to detect the protein levels of BAG3 and α‐SMA. *P < 0.01. Error bars indicate means ± SD

Immunohistochemistry staining demonstrated that BAG3 was expressed in the stoma of some PDAC tissues, accompanied by positive expression of alpha smooth muscle actin (α‐SMA) (Figure 1A). α‐SMA is one of the critical hallmarks of activated PSCs. To ascertain that BAG3 in the extracellular matrix is associated with activated PSCs, in vitro human primary pancreatic stellate cell HPanSteCs were incubated with different concentration of TGF‐β1 for 48 hours. A dose‐dependent increase in mRNA (Figure 1B) and protein (Figure 1C) levels of BAG3 was observed upon HPanSteCs activation, evidenced by an increase of α‐SMA expression levels induced by TGF‐β1 (Figure 1B and C). Activation of HPanSteCs by other factors such as PDGF and IL‐6 also increased BAG3 mRNA (Figure 1D) and protein (Figure 1E) expression levels. To study the potential involvement of BAG3 up‐regulation in activation of PSCs, BAG3 was knocked down using two distinct shRNAs against BAG3 (shBAG3) (Figure 1F). Importantly, knockdown of BAG3 significantly decreased induction of α‐SMA expression by TGF‐β1 (Figure 1F). These data suggested that except for tumour cells, BAG3 was also highly expressed in some activated PSCs in PDAC tissues.

### 3.2 Ectopic BAG3 overexpression promotes proliferation and migration of PSCs

**Figure 2:**
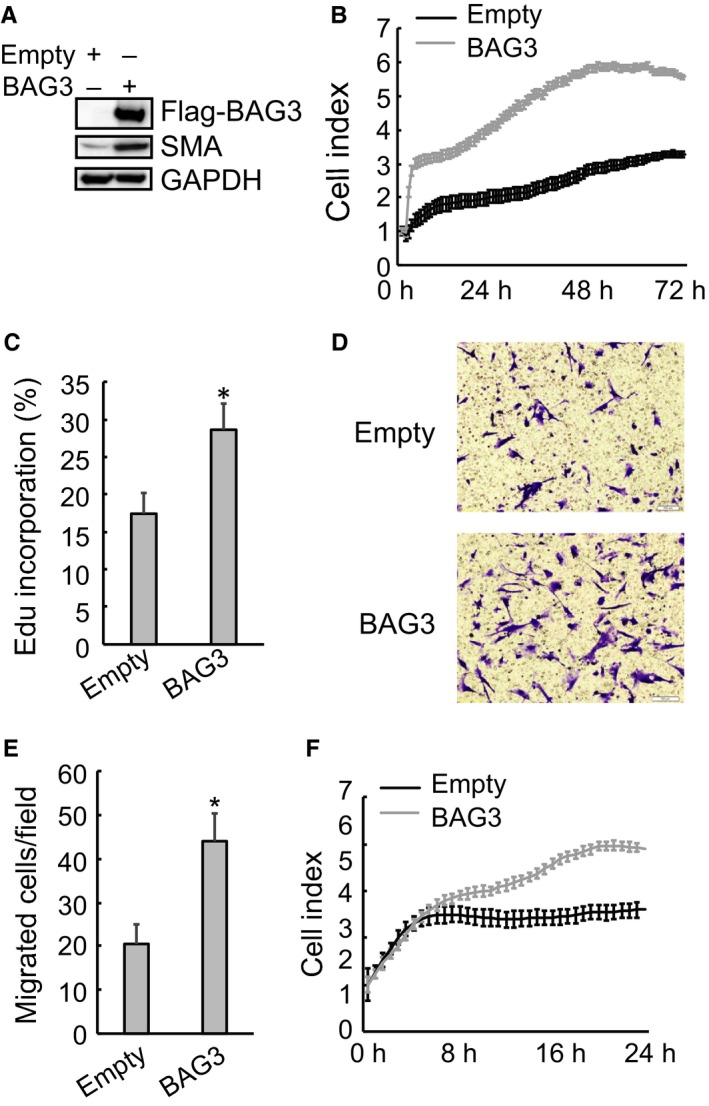
BAG3 overexpression promotes proliferation and migration of HPanSteC cells. (A) HPanSteC cells were infected with lentivirus vectors harbouring BAG3 gene. Western blotting was performed to detect the protein levels of BAG3 and α‐SMA. (B) Control or BAG3‐overexpression HPanSteC cells were seeded on an E plate and real‐time cell indexes were monitored using RTCA. (C) De novo DNA synthesis was analysed using Edu incorporation in control or BAG3‐overexpression HPanSteC cells. (D and E) 3  ×  10^4^ of control or BAG3‐overexpression HPanSteC cells were seeded in serum‐free medium in the upper chamber and migration during 24 hours towards the lower chamber, which contained 10% FBS as a chemoattractant, was evaluated. Migrated cells were counted in 10 randomly chosen high power fields. (F) Control or BAG3‐overexpression HPanSteC cells were seeded on CIM‐plate and real‐time cell indexes were analysed using RTCA. *P < 0.01. Error bars indicate means ± SD

To observe the potential effect of BAG3 on PSCs, HPanSteC cells were infected with lentivirus vectors harbouring BAG3 gene. Western blot analyses indicated that BAG3 elevation in PSCs led to a significant increase in α‐SMA (Figure 2A). RTCA (Figure 2B) and Edu incorporation (Figure 2C) demonstrated that up‐regulation of BAG3 promoted proliferation of HPanSteC cells. Moreover, BAG3 elevation facilitated migration of HPanSteC cells, as assessed by transwell migration assay (Figure 2D) and RTCA analysis (Figure 2E). These data indicated that BAG3 overexpression per se could activate PSCs and promote growth and migration of PSCs.

### 3.3 Conditional media from PSCS with BAG3 overexpression facilitates migration and invasion of PDACS and proliferation and migration of PSCS themselves

**Figure 3:**
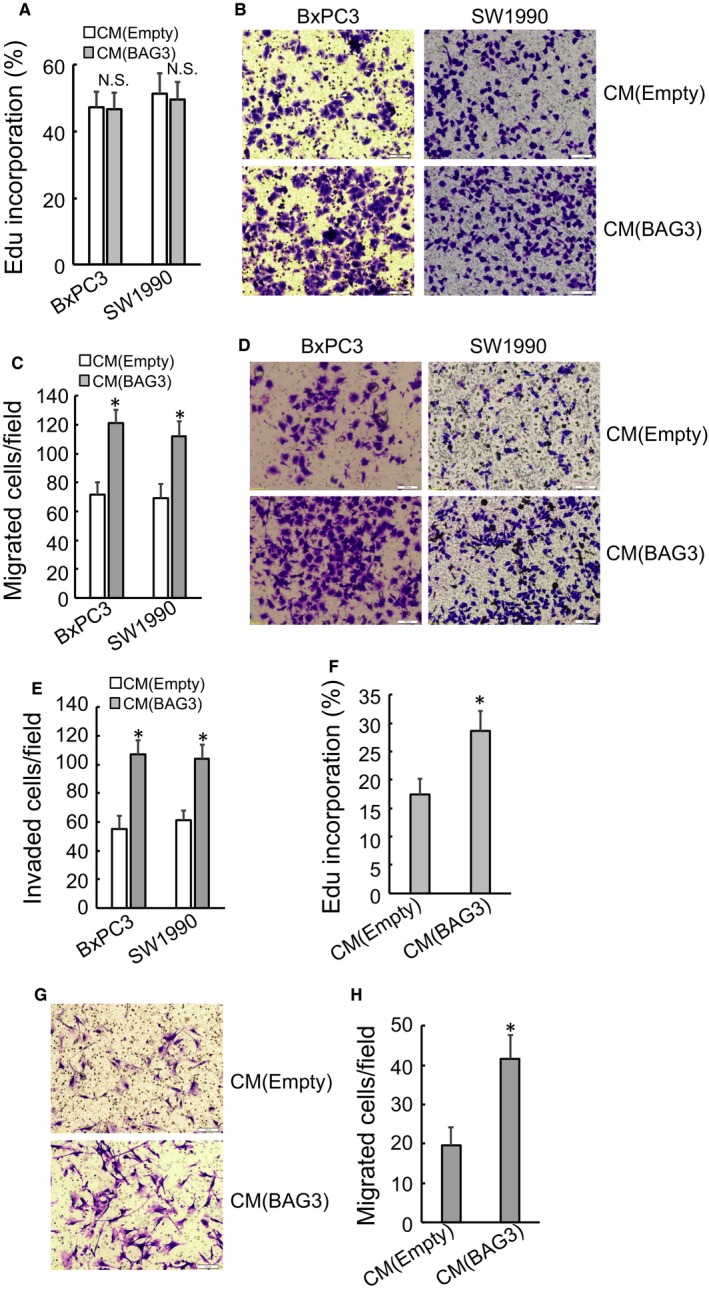
Conditional media from PSCs with BAG3 overexpression affects migration and invasion of PDACs, as well as proliferation and migration of PSCs themselves. (A) BxPC3 and SW1990 cells were treated with conditional media from control or BAG3‐overexpression HPanSteC cells and de novo DNA synthesis was analysed using Edu incorporation. (B and C) Transwell migration assay was performed in BxPC3 and SW1990 cells incubated with conditional media from control or BAG3‐overexpression HPanSteC cells. (D and E) Transwell invasion assay was performed in BxPC3 and SW1990 cells treated with conditional media from control or BAG3‐overexpression HPanSteC cells. (F) HPanSteC cells were treated with conditional media from control or BAG3‐overexpression HPanSteC cells and de novo DNA synthesis was determined using Edu incorporation. (G and H) Transwell migration assay was performed in HPanSteC cells incubated with conditional media from control or BAG3‐overexpression HPanSteC cells. *P < 0.01. Error bars indicate means ± SD

Activated PSCs can exert profound impact on PDACs through secretion of various pro‐inflammatory cytokines/growth factors. Therefore, the influence of BAG3 elevation in PSCs on PDACs was then explored. Edu incorporation assays found that proliferation of two different human PDAC cell lines, BxPC3 and SW1990, was not affected by conditioned media from BAG3‐overexpressed HPanSteC cells, when compared with those from control HPanSteC cells (Figure 3A). However, migration (Figure 3B‐C) and invasion (Figure 3D‐E) of PDAC cell lines were enhanced by addition of conditioned medium from BAG3‐overexpressed HPanSteC cells. Interestingly, conditioned medium from BAG3‐overexpressed HPanSteC cells promoted proliferation (Figure 3F) and migration (Figure 3G‐H) of control HPanSteC cells, as indicated by Edu incorporation and transwell migration assay respectively. These results implied us that BAG3‐positive PSCs might release some factors that promote PDACs invasion and sustain PSCs activation.

### 3.4 Ectopic BAG3 overexpression changes secretory profile of PSCs

**Figure 4:**
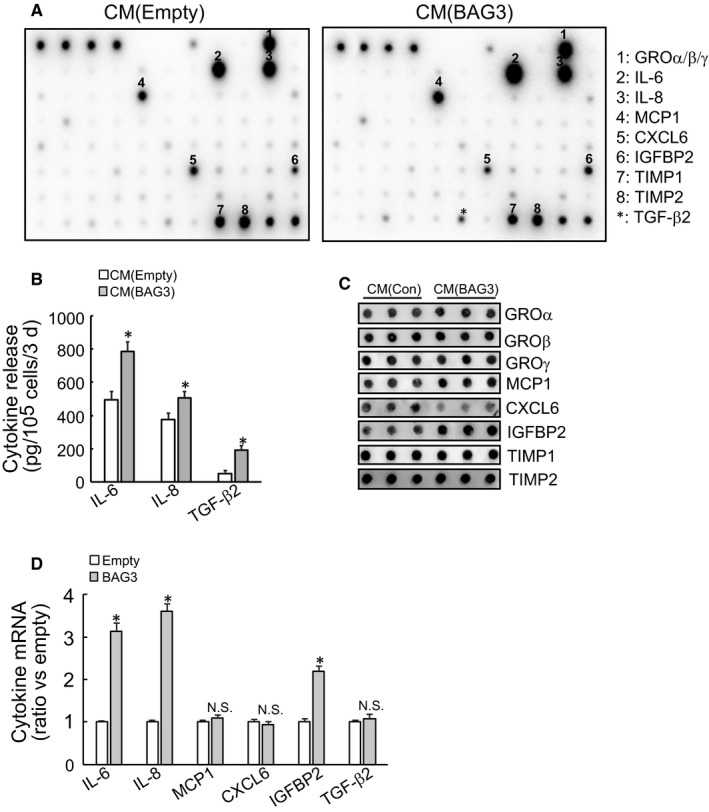
BAG3 overexpression changes secretory profile of PSCs. (A) The conditional media were collected from control or BAG3‐overexpression HPanSteC cultures. These media were used to analyse secretory profile with RayBio® Human Cytokine Antibody Array C‐Series C5 (RayBiotech, USA). (B) The levels of IL‐6, IL‐8 and TGF‐β2 in the conditional media from control or BAG3‐overexpression HPanSteC cells were measured by ELISA. (C) Dot blot was carried out in the conditional media from control or BAG3‐overexpression HPanSteC cells using the indicated antibodies. (D) RT‐qPCR was performed to analyse the mRNA levels of IL‐6, IL‐8, MCP1, CXCL6, IGFBP2 and TGF‐β2 in control or BAG3‐overexpression HPanSteC cells. *P < 0.01. Error bars indicate means ± SD

To identify key factors potentially involved in promotion of PDACs invasion and maintenance of PSCs activation, cytokine protein microarray was performed and identified that secretory levels of interleukin‐6 (IL‐6), IL‐8, Monocyte chemoattractant protein‐1 (MCP‐1), TGF‐β2 and IGFBP2 were markedly increased in conditioned media from BAG3‐ovexpression HPanSteC cells, while CXCL6 was decreased, compared to those from control partners (Figure 4A). GRO α/β/γ and TIMP1/2 were also plentifully secreted by HPanSteC cells, while unaltered by BAG3 overexpression (Figure 4A). Commercial ELISA kits confirmed increases in release of IL‐6, IL‐8 and TGF‐β2 by HPanSteC cells with ectopic BAG3 expression (Figure 4B). Release of MCP1, IGFBP2 and CXCL6 was confirmed by dot blot analyses (Figure 4C). Then real‐time PCR was used to determine mRNA level of key factors. RT‐qPCR demonstrated that BAG3 increased IL‐6, IL‐8 and IGFBP2 mRNA levels, while had no effect on MCP1, CXCL6 or TGF‐β2 mRNA expression (Figure 4D). The results suggested that complicated mechanisms might underlie altering secretory profile of PSCs by BAG3 up‐regulation.

### 3.5 Implication of IL‐6, TGF‐β2 and IGFBP2 in maintenance of HPanSteC activation by BAG3 in an autocrine manner

**Figure 5:**
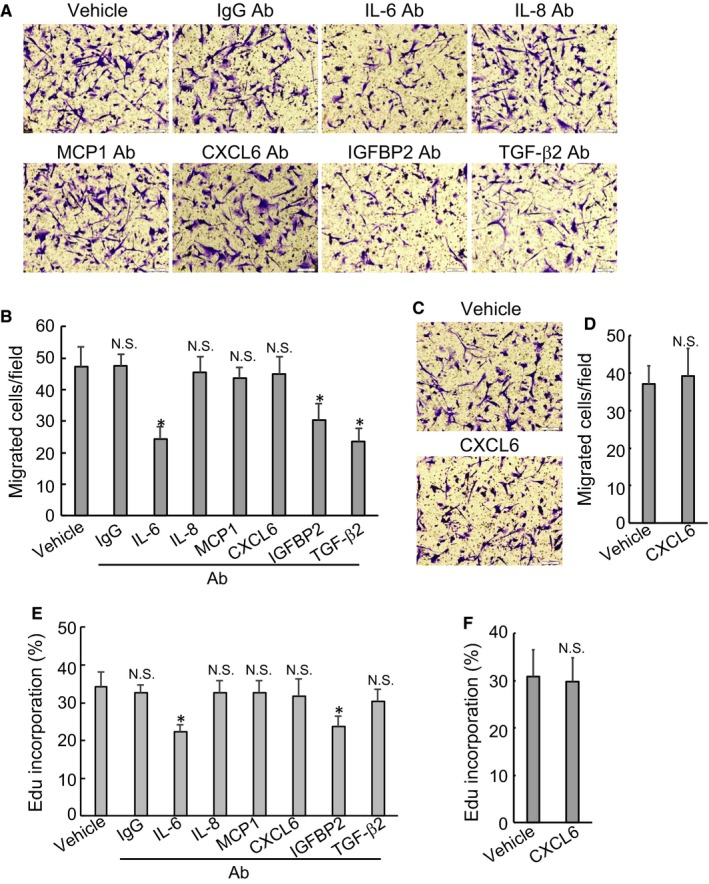
IL‐6, TGF‐β2 and IGFBP2 are involved in maintenance of HPanSteC activation by BAG3 in an autocrine manner. (A and B) The conditional media from BAG3‐overexpression HPanSteC cells were collected and used to treat HPanSteC in the presence of BSA or the indicated monoclonal antibodies. Transwell migration assay was performed in the above cells. (C and D) HPanSteC cells were incubated with conditional media from BAG3‐overexpression HPanSteC cells in the presence of BSA or recombinant CXCL6 (reCXCL6). Transwell migration assay was performed in the above cells. (E and F) HPanSteC cells were incubated with conditional media from BAG3‐overexpression HPanSteC cells in the presence of BSA or the indicated monoclonal antibodies or reCXCL6. Edu incorporation experiments were performed in the above cells. *P < 0.01. Error bars indicate means ± SD

To explore the potential involvement of secretory factors in sustaining PSCs activation, blocking experiments using the indicated antibodies were performed. Addition of IL‐6, TGF‐β2 or IGFBP2 antibody in the culture media significantly suppressed migratory capacity of HPanSteC cells with ectopic BAG3 expression (Figure 5A‐B). Antibodies against IL‐8, MCP1 and CXCL6 exerted no obvious influence on migration of BAG3‐overexpresed HPanSteC cells (Figure 5A‐B). As CXCL6 release was decreased in HPanSteC cells with BAG3 overexpression (Figure 4A,C), recombinant CXCL6 (reCXCL6) was then included in the culture media. Compared with bovine serum albumin (BSA), reCXCL6 did not alter migration of HPanSteC cells (Figure 5C‐D). Edu incorporation experiments demonstrated that proliferation of BAG3‐overexpressed HPanSteC cells was inhibited by addition of IL‐6 and IGFBP2 antibodies, while other antibodies had no obvious effects (Figure 5E). Neither reCXCL6 affected proliferation of BAG3‐overexpressed HPanSteC cells (Figure 5F). These data indicated that BAG3 might promote release of IL‐6, TGF‐β2 and IGFBP2 by PSCs to sustain their own activation.

### 3.6 BAG3‐positive HPanSteC cells promotes PDACs invasion via IL‐8, MCP1, TGF‐β2 and IGFBP2

**Figure 6:**
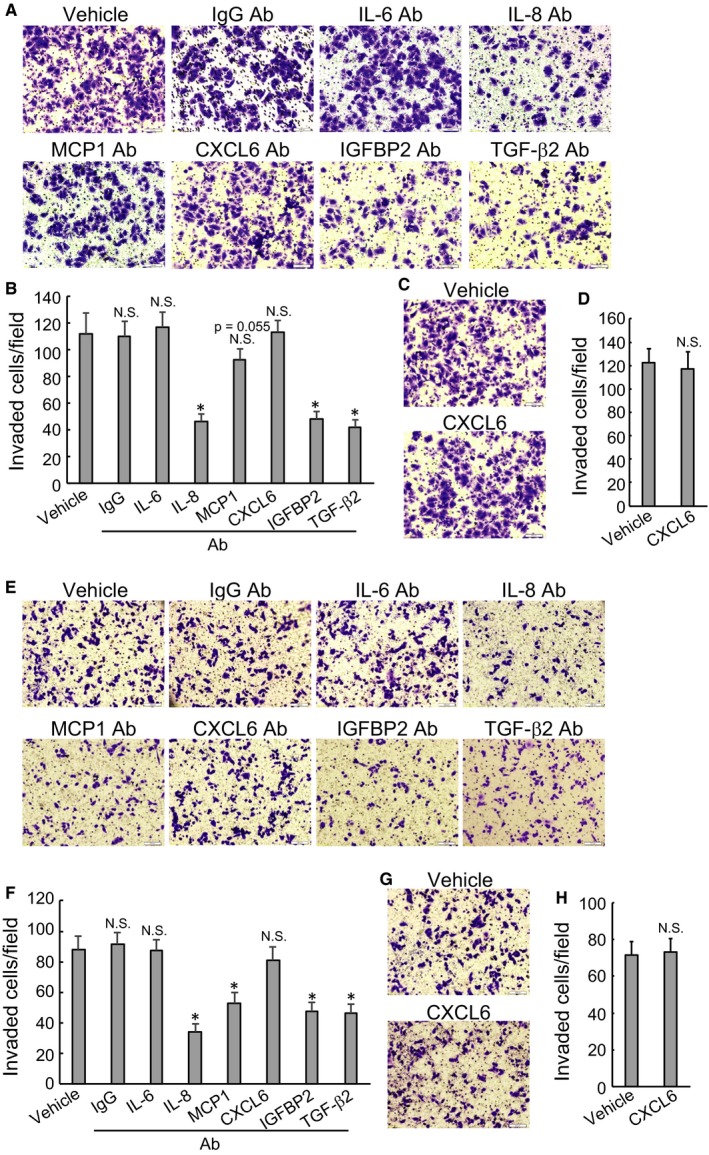
BAG3‐positive HPanSteC cells promote PDACs invasion via IL‐8, MCP1, TGF‐β2 and IGFBP2 release. (A, B, E and F) BxPC3 (A and B) or SW1990 cells (E and F) were incubated with conditional media from BAG3‐overexpression HPanSteC cells in the presence of BSA or the indicated monoclonal antibodies. Transwell invasion assay was performed in the above cells. (C, D, G and H) BxPC3 (C and D) or SW1990 (G and H) cells were incubated with conditional media from BAG3‐overexpression HPanSteC cells in the presence of BSA or reCXCL6. Transwell invasion assay was performed in the above cells. *P < 0.01. Error bars indicate means ± SD

To elucidate the potential implication of secretory factors from PSCs in promoting invasion of PDACs, neutralizing antibodies were supplemented to the conditioned media collected from HPanSteC cells with ectopic BAG3 expression. Invasion of BxPC3 cells was significantly suppressed by antibodies against IL‐8, IGFBP2 and TGF‐β2 (Figure 6A‐B). Neither CXCL6 antibody (Figure 6A‐B) nor recombinant CXCL6 (Figure 6C‐D) altered invasive capacity of BxPC3 cells. Similarly, IL‐8, IGFBP2, TGF‐β2 blocking also suppressed invasion of SW1990 cells (Figure 6E‐F). In addition, MCP1 blocking also suppressed the effect of conditioned medium from BAG3‐overexpressed PSCs on SW1990 invasion of PDAC cell line (Figure 6E‐F). Neither CXCL6 antibody (Figure 6E‐F) nor recombinant CXCL6 (Figure 6G‐H) exerted effects on invasion of SW1990 cells.

## 4 DISCUSSION

BAG3 is up‐regulated in cancer cells and its overexpression is correlated with tumour progression and poor prognosis of PDAC.^22^, ^23^ Recent study has demonstrated that BAG3 can be secreted by PDAC cells and secreted BAG3 promotes pancreatic ductal adenocarcinoma proliferation via activating stromal macrophages in tumour microenvironment.^20^ Previous reports have mainly highlighted the oncogenic role of BAG3 highly expressed by cancer cells themselves. The current study demonstrates that BAG3 produced by stromal cells plays a different role, focusing on tumour microenvironment. In this study, we observed that beside in PDACs, BAG3 was also significantly expressed in the stroma of some PDAC tissues. In vitro, we found that BAG3 was hardly expressed in quiescent PSCs, while de novo expression of BAG3 was marked induced once PSCs were activated by TGF‐β1, PDGF and IL‐6. Importantly, knockdown of BAG3 decreased the extent of PSCs activation induced by TGF‐β1, indicating that BAG3 at least partially implicated in PSCs activation. Furthermore, ectopic expression of BAG3 through lentivirus infection could activate PSCs, evidenced by elevated α‐SMA expression. Besides, ectopic BAG3 overexpression directly stimulated proliferation and migration of PSCs. These data suggest that BAG3‐positive PSCs might play a potential role in remodelling of tumour microenvironment in PDAC.

It is well known that tumour microenvironment plays a pivotal role in cancer progression, metastasis and chemotherapy resistance.^24^, ^25^ The pancreatic tumour microenvironment is comprised of various cells including stellate cells, fibroblast, immune cells, as well as blood vessels, extracellular matrix proteins.^26^ The interaction between tumour microenvironment and PDAC cells has extensively been demonstrated.^4^ PSCs, one of important cellular components in stroma of PDAC, exist in two main forms: quiescent and activated.^27^ Quiescent PSCs have key function in regulation of extracellular matrix turnover and in maintenance of normal tissue architecture.^27^, ^28^ On the contrast, activated PSCs play a central role in PDAC growth, evasion of immune surveillance, invasion, metastasis and resistance to chemotherapy.^29^, ^30^, ^31^, ^32^ Interestingly, the current study showed that conditioned media from BAG3‐overexpression PSCs facilitate migration and invasion of PDACs, implying that BAG3 may interlink PDACs and PSCs via regulating secretion function of PSCs. Recent reports have shown that activated PSCs can secret various soluble factors, such as TGF‐β, IL‐6 and SDF‐1, to regulate PDAC cell proliferation, migration, epithelial‐mesenchymal transition (EMT), chemoradiation resistance and/or sustain activated status of themselves.^33^ The current study demonstrated that ectopic BAG3 overexpression increased secretion of several factors, including IL‐6, IL‐8, MCP1, TGF‐β2 and IGFBP2 in PSCs. Further neutralizing antibodies experiments demonstrated that BAG3‐positive PSCs promote PDACs invasion via releasing IL‐8, MCP1, TGF‐β2 and IGFBP2. In addition to influence on PDACs, BAG3 also sustains PSCs in activated status by elevating IL‐6, TGF‐β2 and IGFBP2. These cytokines and growth factors may act via autocrine signalling as other studies have reported.^34^ Sustained activation of PSCs can disrupt the biomechanical balance of nearby microenvironments, thus contributing to invasion of PDACs.^35^ Thus, BAG3‐positive PSCs remodel tumour microenvironment favourable for migration and invasion of cancer cells through multiple key cytokines/chemokines.

BAG3 might regulate the subset of cytokines/chemokines expression via distinct mechanisms. Although BAG3 changed release of IL‐6, IL‐8, MCP1, CXCL6, IGFBP2 and TGF‐β2 by PSCs, it exerted no obvious effect on MCP1, CXCL6, TGF‐β2 mRNAs. These discordant mRNA and protein expression can be caused by many contributors. First of all, regulation of gene expression in eukaryotes involves many steps including transcription initiation, post‐transcriptional regulation, translation and degradation of the protein. BAG3 achieves a variety of oncogenic functions by interaction with various protein partners. For example, through interaction with the heat shock protein HSP70, BAG3 could modulate the activity of many proteins, including the transcription factors NF‐κB, FoxM1, Hif1α, the translation regulator HuR and the cell‐cycle regulators p21 and survivin.^36^ Thereby BAG3 might regulate some genes expression at the transcriptional activation step via modulation of their specific transcriptional factors. In addition, recent studies have shown that BAG3 possesses RNA binding motifs and function as a RNA binding protein.^37^, ^38^ Thereby, BAG3 might also regulate some genes expression at the post‐transcriptional and translational levels via interaction with target transcripts. Alternatively, we detected the secretion levels of the above cytokines and growth factors; we cannot exclude the possibility that BAG3 might regulate release of cytokines and growth factors by regulating cytoskeleton. A previous study reported that BAG3 could regulate insulin secretion.^39^ The exact mechanisms by which BAG3 regulates secretion of multiple key protein factors in PSCs need further investigation.

**Figure 7:**
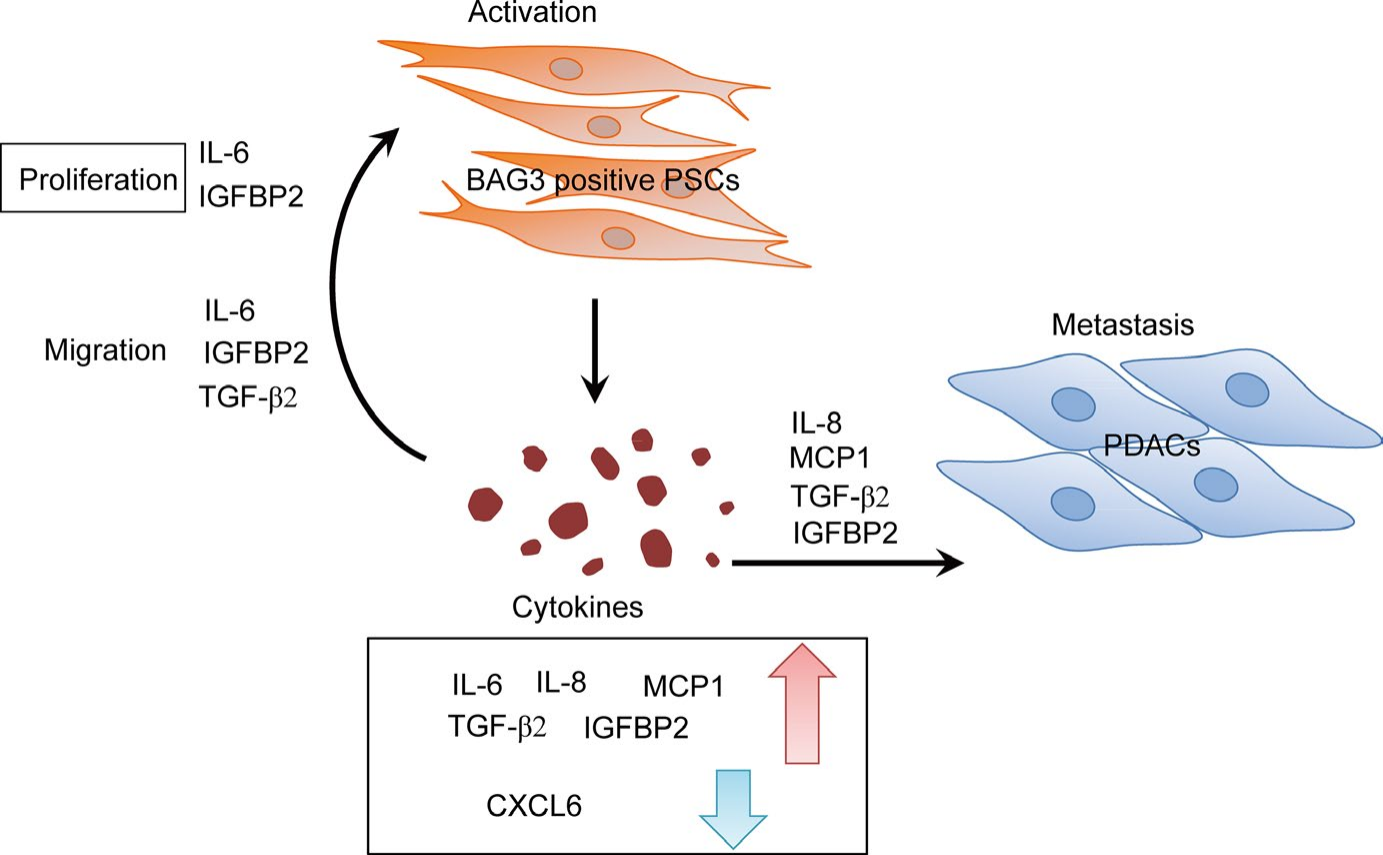
Schematic representation of interplay between PSCs and PDACs mediated by BAG3 expression in PSCs. Briefly, BAG3‐positive PSCs secretes high levels of multiple cytokines/chemokines including IL‐6, IL‐8, MCP1, IGFBP2 and TGF‐β2. On one hand, IL‐8, MCP1, IGFBP2 and TGF‐β2 contribute to migration and invasion of PDACs in a paracrine manner. On the other hand, IL‐6, TGF‐β2 and IGFBP2 in turn maintain HPanSteC persistent activation in an autocrine manner. Thereby BAG3‐positive PSCs remodel tumour microenvironment favouring malignant progression of PDACs

In conclusion, the current study demonstrated that BAG3 expressed in PSCs maintains their own activation and promotes migration and invasion of pancreatic cancer cells via autocrine and paracrine respectively. On one hand, BAG3 promotes release of soluble protein factors including IL‐6, TGF‐β2, IGFBP2, which in turn act on themselves to sustain PSCs activation (Figure 7). On the other hand, BAG3‐positive PSCs also facilitate migration and invasion of nearby pancreatic cancer cells via secretion of soluble protein factors including IL‐8, MCP1, TGF‐β2 and IGFBP2 (Figure 7). Thereby, interplay between PSCs and PDACs meditated by BAG3 establishes a niche which contributes to migration and invasion of PDACs. Based on the fact that BAG3 has an obvious influence on microenvironment of PDACs, targeting BAG3 might relieve desmoplasia, thereby delaying migration and invasion of PDACs.

## CONFLICT OF INTEREST

The authors declared there are no competing financial interests.

## References

[1] Siegel RL , Miller KD , Jemal A . Cancer Statistics, 2017. CA Cancer J Clin. 2017;67:7‐30. 28055103 10.3322/caac.21387

[2] Mayor S . Deaths from pancreatic cancer in Europe continue to increase while rates for other cancers fall. BMJ. 2014;348:g2914. 24764567 10.1136/bmj.g2914

[3] Ying H , Dey P , Yao W , et al. Genetics and biology of pancreatic ductal adenocarcinoma. Genes Dev. 2016;30:355‐385. 26883357 10.1101/gad.275776.115 PMC4762423

[4] Erkan M , et al. The role of stroma in pancreatic cancer: diagnostic and therapeutic implications. Nat Rev Gastroenterol Hepatol. 2012;9:454‐467. 22710569 10.1038/nrgastro.2012.115

[5] Neesse A , Algul H , Tuveson DA , Gress TM . Stromal biology and therapy in pancreatic cancer: a changing paradigm. Gut. 2015;64:1476‐1484. 25994217 10.1136/gutjnl-2015-309304

[6] Waghray M , Yalamanchili M , di Magliano MP , Simeone DM . Deciphering the role of stroma in pancreatic cancer. Curr Opin Gastroenterol. 2013;29:537‐543. 23892539 10.1097/MOG.0b013e328363affe PMC4112589

[7] Erkan M , Michalski CW , Rieder S , et al. The activated stroma index is a novel and independent prognostic marker in pancreatic ductal adenocarcinoma. Clin Gastroenterol Hepatol. 2008;6:1155‐1161. 18639493 10.1016/j.cgh.2008.05.006

[8] Neesse A , Michl P , Frese KK , et al. Stromal biology and therapy in pancreatic cancer. Gut. 2011;60:861‐868. 20966025 10.1136/gut.2010.226092

[9] Korc M . Pancreatic cancer‐associated stroma production. Am J Surg. 2007;194:S84‐86. 17903452 10.1016/j.amjsurg.2007.05.004 PMC2094116

[10] Chu GC , Kimmelman AC , Hezel AF , DePinho RA . Stromal biology of pancreatic cancer. J Cell Biochem. 2007;101:887‐907. 17266048 10.1002/jcb.21209

[11] Kota J , Hancock J , Kwon J , Korc M . Pancreatic cancer: Stroma and its current and emerging targeted therapies. Cancer Lett. 2017;391:38‐49. 28093284 10.1016/j.canlet.2016.12.035

[12] Mei L , Du W , Ma WW . Targeting stromal microenvironment in pancreatic ductal adenocarcinoma: controversies and promises. J Gastrointest Oncol. 2016;7:487‐494. 27284483 10.21037/jgo.2016.03.03 PMC4880763

[13] Özdemir B , Pentcheva‐Hoang T , Carstens J , et al. Depletion of carcinoma‐associated fibroblasts and fibrosis induces immunosuppression and accelerates pancreas cancer with reduced survival. Cancer Cell. 2014;25:719‐734. 24856586 10.1016/j.ccr.2014.04.005 PMC4180632

[14] Rhim A , Oberstein P , Thomas D , et al. Stromal elements act to restrain, rather than support, pancreatic ductal adenocarcinoma. Cancer Cell. 2014;25:735‐747. 24856585 10.1016/j.ccr.2014.04.021 PMC4096698

[15] Rosati A , Graziano V , De Laurenzi V , Pascale M , Turco MC . BAG3: a multifaceted protein that regulates major cell pathways. Cell Death Dis. 2011;2:e141. 21472004 10.1038/cddis.2011.24 PMC3122056

[16] Rosati A , Bersani S , Tavano F , et al. Expression of the antiapoptotic protein BAG3 is a feature of pancreatic adenocarcinoma and its overexpression is associated with poorer survival. Am J Pathol. 2012;181:1524‐1529. 22944597 10.1016/j.ajpath.2012.07.016

[17] Guerriero L , et al. BAG3 protein expression in melanoma metastatic lymph nodes correlates with patients' survival. Cell Death Dis. 2014;5:e1173. 24722298 10.1038/cddis.2014.143 PMC5424119

[18] Yang X , et al. Bag‐3 expression is involved in pathogenesis and progression of colorectal carcinomas. Histol Histopathol. 2013;28:1147‐1156. 23553442 10.14670/HH-28.1147

[19] Chiappetta G , Ammirante M , Basile A , et al. The antiapoptotic protein BAG3 is expressed in thyroid carcinomas and modulates apoptosis mediated by tumor necrosis factor‐related apoptosis‐inducing ligand. J Clin Endocrinol Metab. 2007;92:1159‐1163. 17164298 10.1210/jc.2006-1712

[20] Rosati A , et al. BAG3 promotes pancreatic ductal adenocarcinoma growth by activating stromal macrophages. Nat Commun. 2015;6:8695. 26522614 10.1038/ncomms9695 PMC4659838

[21] Falco A , Rosati A , Festa M , et al. BAG3 is a novel serum biomarker for pancreatic adenocarcinomas. Am J Gastroenterol. 2013;108:1178‐1180. 23821002 10.1038/ajg.2013.128

[22] Liao Q , Ozawa F , Friess H , et al. The anti‐apoptotic protein BAG‐3 is overexpressed in pancreatic cancer and induced by heat stress in pancreatic cancer cell lines. FEBS Lett. 2001;503:151‐157. 11513873 10.1016/s0014-5793(01)02728-4

[23] Shi H , et al. BAG3 promotes chondrosarcoma progression by upregulating the expression of beta‐catenin. Mol Med Rep. 2018;17:5754‐5763. 29484408 10.3892/mmr.2018.8611 PMC5866018

[24] Klemm F , Joyce JA . Microenvironmental regulation of therapeutic response in cancer. Trends Cell Biol. 2015;25:198‐213. 25540894 10.1016/j.tcb.2014.11.006 PMC5424264

[25] Quail DF , Joyce JA . Microenvironmental regulation of tumor progression and metastasis. Nat Med. 2013;19:1423‐1437. 24202395 10.1038/nm.3394 PMC3954707

[26] Ansari D , Friess H , Bauden M , Samnegard J , Andersson R . Pancreatic cancer: disease dynamics, tumor biology and the role of the microenvironment. Oncotarget. 2018;9:6644‐6651. 29464100 10.18632/oncotarget.24019 PMC5814240

[27] Apte MV , Pirola RC , Wilson JS . Pancreatic stellate cells: a starring role in normal and diseased pancreas. Front Physiol. 2012;3:344. 22973234 10.3389/fphys.2012.00344 PMC3428781

[28] Means AL . Pancreatic stellate cells: small cells with a big role in tissue homeostasis. Lab Invest. 2013;93:4‐7. 23269285 10.1038/labinvest.2012.161

[29] Apte MV , Wilson JS . Dangerous liaisons: pancreatic stellate cells and pancreatic cancer cells. J Gastroenterol Hepatol. 2012;27(Suppl 2):69‐74. 10.1111/j.1440-1746.2011.07000.x 22320920

[30] Hamada S , Masamune A , Takikawa T , et al. Pancreatic stellate cells enhance stem cell‐like phenotypes in pancreatic cancer cells. Biochem Biophys Res Comm. 2012;421:349‐354. 22510406 10.1016/j.bbrc.2012.04.014

[31] Uchida M , Ito T , Nakamura T , et al. Pancreatic stellate cells and CX3CR31: occurrence in normal pancreas and acute and chronic pancreatitis and effect of their activation by a CX3CR31 agonist. Pancreas. 2014;43:708‐719. 24681877 10.1097/MPA.0000000000000109 PMC4315317

[32] Zhang H , et al. Paracrine SDF‐1alpha signaling mediates the effects of PSCs on GEM chemoresistance through an IL‐6 autocrine loop in pancreatic cancer cells. Oncotarget. 2015;6:3085‐3097. 25609203 10.18632/oncotarget.3099 PMC4413639

[33] Wu QI , Tian Y , Zhang J , et al. Functions of pancreatic stellate cell‐derived soluble factors in the microenvironment of pancreatic ductal carcinoma. Oncotarget. 2017;8:102721‐102738. 29254283 10.18632/oncotarget.21970 PMC5731993

[34] Wilson JS , Pirola RC , Apte MV . Stars and stripes in pancreatic cancer: role of stellate cells and stroma in cancer progression. Front Physiol. 2014;5:52. 24592240 10.3389/fphys.2014.00052 PMC3924046

[35] Zhan H‐X , Zhou B , Cheng Y‐G , et al. Crosstalk between stromal cells and cancer cells in pancreatic cancer: new insights into stromal biology. Cancer Lett. 2017;392:83‐93. 28189533 10.1016/j.canlet.2017.01.041

[36] Colvin TA , Gabai VL , Gong J , et al. Hsp70‐Bag3 interactions regulate cancer‐related signaling networks. Can Res. 2014;74:4731‐4740. 10.1158/0008-5472.CAN-14-0747 PMC4174322 24994713

[37] An M‐X , Li SI , Yao H‐B , et al. BAG3 directly stabilizes Hexokinase 2 mRNA and promotes aerobic glycolysis in pancreatic cancer cells. J Cell Biol. 2017;216:4091‐4105. 29114069 10.1083/jcb.201701064 PMC5716268

[38] Rodríguez AE , López‐Crisosto C , Peña‐Oyarzún D , et al. BAG3 regulates total MAP1LC3B protein levels through a translational but not transcriptional mechanism. Autophagy. 2016;12:287‐296. 26654586 10.1080/15548627.2015.1124225 PMC4836015

[39] Iorio V , Festa M , Rosati A , et al. BAG3 regulates formation of the SNARE complex and insulin secretion. Cell Death Dis. 2015;6:e1684. 25766323 10.1038/cddis.2015.53 PMC4385931

